# Supplementary material for: The Role of Active Packaging in the Defense Against Foodborne Pathogens with Particular Attention to Bacteriophages
Source: Microorganisms. 2025 Feb 12;13(2):401. doi: 10.3390/microorganisms13020401 (PMC11858251; doi:10.3390/microorganisms13020401)
Supplement: Supplementary file 1 [file microorganisms-13-00401-s001.zip › microorganisms-3307795-supplementary.pdf]

Table S1. Commercially available bacteriophages and company related worldwide web addresses.

Trade name of phage product	Targeted microbes/cocktail	Company	Country	Application	Website
ProBe-Bac PE®	Cocktail	©Pathway Intermediates	Republic of Korea	Poultry multi-bacterial infections.	http://www.pathway-intermediates.com
ProBe-Bac SE®				Swine multi-bacterial infections	
AP-PA02®	*Pseudomonas aeruginosa*	©Armata Pharmaceuticals, Inc.	USA	Therapeutic applications	https://www.armatapharma.com/
AP-PA03®					
AP-SA02®	*Staphylococcus aureus*				
ListShield™ (GRAS)	*Listeria monocytogenes*	©Intralytix, Inc	USA	For controlling *Listeria monocytogenes* infections	http://www.intralytix.com/index.php
SalmoFresh™ (GRAS)	*Salmonella* spp.			For controlling *Salmonella spp.* infection	
EcoShield™ PX	*E. coli* (STEC); O157: H7 STEC.			Controlling the foodborne *Escherichia coli* infection	
ShigaShield™ (GRAS)	*S. flexneri, S. sonnei and S. dysenteriae.*			Effective Against *Shigella* spp.	
CampyShield™	*Campylobacter jejuni*			Controlling the foodborne/waterborne bacterial pathogen Campylobacter.	
SalmoLyse®	*Salmonella* spp.			Controlling the foodborne bacterial pathogen *Salmonella enterica* in pet food.	
PhageGuard Listex® (GRAS)	*Listeria* spp.	©Micreos BV	Netherlands	Controlling the foodborne bacterial pathogen *Listeria* spp.	https://phageguard.com/
PhageGuard S.® (GRAS)	*Salmonella* spp.			Against foodborne *Salmonella* spp.	
PhageGuard E.® (GRAS)	*E. coli*			Against foodborne *E. coli*	

LUXON®	*Vibrio parahaemolyticus*	©Phagelux (Nanjing) Biotech Company Limited	China	Control early mortality syndrome (EMS, or AHPND) in shrimps caused by *Vibrio parahaemolyticus*.	http://www.phageluxagrihealth.com/en/
LUNIN®	*Staphylococcus, E. coli, P. aeruginosa or Salmonella*			To control *Staphylococcus*, *E. coli*, *P. aeruginosa*, or *Salmonella* infections in chicken farms	
LUMON®	*Staphylococcus, E. coli, P. aeruginosa, Salmonella or K. pneumoniae*			*Staphylococcus, E. coli, P. aeruginosa, Salmonella,* or *K. pneumonia* infections in cattle farms	
LUZON®	*Staphylococcus, E. coli, P. aeruginosa or Salmonella*			*Staphylococcus*, *E. coli*, *P. aeruginosa*, or *Salmonella* infections in pig farms	
SHIJUNSHA®	*Staphylococcus, E. coli, P. aeruginosa and Salmonella*			To control salpingitis & peritonitis in poultry breeding environment	
AgriPhage - Fire ®Blight	*Erwinia amylovora*			Agricultural bactericide	
AgriPhage-Citrus Canker™	*Xanthomonas citri subsp. Citri*			Agricultural bactericide	
Secure Shield E1®	*Escherichia Coli*	@FINK TEC GmbH	Germany	Food safety applications.	https://www.finktec.com/applied-phage
Biotector® Poultry	*Salmonella, C. perfringens and Escherichia coli.*	©Cheiljedang Corp.	Republic of Korea	Bacteriophage cocktail of animal feed additive.	https://www.cjbio.net/en/products/biotector.do
Biotector® Swine	*Escherichia coli*				
Finalyse™	*E. coli* O157: H7	©Church & Dwight Co., Inc	USA	To control *E. coli*	https://ahfoodchain.com/
Finalyse SAL™	*Salmonella* spp.			To reduce *Salmonella* in poultry processing.	
BX004®	*P. aeruginosa*	©BiomX	USA	Therapy for respiratory infections with *Pseudomonas aeruginosa*	https://www.biomx.com/
BX003®	*Klebsiella pneumoniae*			Therapeutic application	

BX005®	*Staphylococcus aureus*				
Colorectal cancer-based phage	*Fusobacterium nucleatum*			Colorectal cancer therapy	
Pyo-Phage®	*Staphylococcus, E. coli, Streptococcus, Pseudomonas, Proteus*				
EnkoPhagum®	*Salmonellae [Paratyphus A, Paratyphus B, Typhimurium, Enteritidis, Choleraesuis, Oranienburg, Dublin, Anatum], Shigellas [Flexner, Zonne], Enteropathogenic serotypes of Escherichia coli* [10 types], *Staphylococcus* [3 types]				
SES Phage®	*Staphylococcus phage, Streptococcus phage, E.coli*	©Brimrose Technology Corporation	USA	Animal feed-related products	https://www.brimrosetechnology.com
Intesti Phage®	*Salmonella spp., Staphylococcus, Cholera suis, P.aeruginosa Proteus spp., E.coli – different serotypes Shigella spp.,,*				
Fersisi Phage®	*Staphylococcus phage* [3 types] *Streptococcus phage* [4 types]				
Mono-phage® Preparations	*Staphylococcal, E. coli, Streptococcal, Pseudomonas aeruginosa, Proteus*				
Pherecydes®	*Staphylococcus aureusphages,*	© Pherecydes Pharma	USA	Therapeutic application	https://www.pherecydes-pharma.com/

	Pseudomonas aeruginosa, Escherichia coli				
N-Rephasin® SAL200					
EL200®					
TL200®					
SJ200®	Infectious diseases	© iNtRON Biotechnology, Inc.	Republic of Korea	Antibacterial, antifungal, multiple drug resistance	https://intodeworld.com/
SEL-2®					
CH200®					
EYD34®					
ASPject PT3.9	*Pseudomonas aeruginosa*				
SASPject™ PT4	*Klebsiella pneumoniae*	© Phico Therapeutics Limited.	UK	Therapeutic application	https://phicotx.co.uk/#products
SASPject™ PT5	*Escherichia coli*				
SASPject™ PT1.2	*Staphylococcus aureus*				
Otophage©	Cocktails	© RPC, Micromir	Russia	Normalization of microflora in ENT organs (Human)	https://micromir.bio/otophage
Phagodent© (cocktail)	*Acinetobacter baumannii* *Aggregatibacter actinomycetemcomitans* *Bacillus licheniformis* *Bacteroides fragilis* *Cutibacterium acnes (Propionibacterium acnes)*	© RPC, Micromir	Russia	Presentation of development of inflammatory diseases in the oral cavity. (Human)	https://micromir.bio/phagodent

	Enterobacter cloacae<br>*Enterobacter kobei*<br>*Enterococcus faecalis*<br>*Enterococcus faecium*<br>*(Streptococcus groups D)*<br>*Klebsiella pneumoniae*<br>*Klebsiella pneumoniae subsp. ozaenae*<br>*Proteus mirabilis*<br>*Proteus vulgaris*<br>*Pseudomonas aeruginosa*<br>*Staphylococcus aureus*<br>*Staphylococcus capitis*<br>*Staphylococcus caprae*<br>*Staphylococcus epidermidis*<br>*Staphylococcus haemolyticu*<br>*Staphylococcus warneri*<br>*Stenotrophomonas maltophilia*<br>*Streptococcus agalactiae*<br>*Streptococcus pyogenes*<br>*Wolinella spp.*				
Phagogyn© (cocktail)	Cocktail			Presentation of bacterial infections in the intimate area	https://micromir.bio/phagogyn
Phagoderm© (cocktail)	Cocktail	© RPC, Micromir	Russia	Presentation of bacterial infections of the skin (Human)	https://micromir.bio/phagoderm
Iskraphage©	Cocktail	© RPC, Micromir & Iskra Sangyo Co., Ltd.	Russia/Japan	To prevent bacterial infections of the skin (Human)	https://iskraphage.jp/
Vetagyn© (cocktail)	Cocktail	© RPC, Micromir	Russia	For prevention and treatment of bacterial endometritis (Veterinary)	https://micromir.bio/fagouterin-en
Phagovet©	Cocktail	© RPC, Micromir	Russia	Used to prevent bacterial diseases in farming broilers, rearing flocks, and adult flocks. (Veterinary)	https://micromir.bio/phagovet

aquaPHIX™<br>petPHIX™<br>farmPHIX™<br>mediPHIX™<br>safePHIX™<br>carePHIX™<br>agriPHIX™<br>freshPHIX™	NA<br>Cocktail	©FIXED PHAGE	United Kingdom	Agriculture/livestock applications	https://www.fixed-phage.com//
BAFACOL™	*Escherichia coli*	©Proteon Pharmaceuticals	Poland	To prevent Avian Pathogenic E. coli in poultry.	https://www.proteonpharma.com/
BAFADOR™	Cocktail			Feed additive cocktail for Aquaculture.	
BAFASAL®	Cocktail			Feed additive	
BAFASAL + G®	Cocktail				
PYO Bacteriophage	*Staphylococcus aureus, Streptococcus (S. pyogenes, S. sanguis, S.salivarius and S. agalactiae), Escherichia coli, Pseudomonas aeruginosa and Proteus (P. mirabilis and P. vulgaris).*	©Eliava BioPreparations	USA	Therapeutic application	https://phage.ge
Intesti Bacteriophage	*Shigella flexneri (serotypes 1,2,3,4, S. sonnei, Shigella*				

	Newcastle type), Salmonella (Paratyphi A, B, S. Typhimurium, S. enteritidis, S. choleraesuis and S. oranienburg), Escherichia coli, Proteus (P. vulgaris and P. mirabilis), Staphylococcus aureus, Pseudomonas aeruginosa and Enterococcus faecalis.				
Staphylococcal Bacteriophage	*Staphylococcus aureus.*				
SES Bacteriophage	*Staphylococci, Streptococci, and Enteropathogenic Escherichia coli*				
FERSISI Bacteriophage	*Staphylococci and Streptococci.*				
ENKO Bacteriophage	*Staphylococcus, Shigella, Escherichia coli, and Salmonella*				
Biolyse®–PB	*Pectobacterium spp, Xanthomonas spp. Ralstonia spp.*	©APS Biocontrol Ltd.	UK	Soft-rot bacteria of potatoes.	https://www.apsbiocontrol.com/
PHAGYO	*Streptococcus, Staphylococcus, Escherichia coli, Pseudomonas aeruginosa, Proteus and their combinations.*	©Biochimpharm	Georgia, USA	Therapeutic application	https://biochimpharm.ge/
Septaphage	*Shigella (S. flexneri 1,2,3,4,6 serogroup and S. sonnei), Salmonella (paratyphi A, B, S. Typhimurium, S.*				

	choleraesuis, S. oranienburg, and S. enteritidis), Enteropathogenic Escherichia coli etiologically important serogroups of enteral diseases, Proteus (P. vulgaris and P. mirabilis), Staphylococcus, Pseudomonas and Enterococcus.
PHAGESTAPH	*Staphylococcus aureus.*
Septaphage tablets	*Shigella (S. flexneri 1,2,3,4,6 serogroup and S. sonnei), Salmonella (paratyphi A, B, S. Typhimurium, S. choleraesuis, S. oranienburg and S. enteritidis), Enteropathogenic Escherichia coli – etiologically important serogroups of enteral diseases, Proteus (P. vulgaris and P. mirabilis), Staphylococcus, Pseudomonas and Enterococcus.*
Travelphage capsules	*Shigella, Salmonella, Escherichia coli, Proteus, Staphylococcus, Pseudomonas and Enterococcus*

PreforPro	*Cocktail*	Deerland Probiotics & Enzymes	USA	Prebiotic	https://www.preforpro.com/
